# Review of Strain Rate Effects of Fiber-Reinforced Polymer Composites

**DOI:** 10.3390/polym13172839

**Published:** 2021-08-24

**Authors:** Lulu Ma, Feng Liu, Dongyu Liu, Yaolu Liu

**Affiliations:** 1 Department of Mechanical Engineering, Lamar University, Beaumont, TX 77710, USA; llmtju@163.com; 2 Nanjing Changjiang Waterway Engineering Bureau, No. 9 Jiangbian Road, Nanjing 210011, China; liufeng@njhdgcj.net; 3 ZWSOFT Co., Ltd., No. 15 Zhujiang West Road, Guangzhou 510623, China; liudongyu@zwcad.com; 4 Faculty of Civil Engineering and Geosciences, Delft University of Technology, P.O. Box 5048, 2600 GA Delft, The Netherlands

**Keywords:** composites, strain rate, impact, multiscale, mechanism

## Abstract

The application of fiber-reinforced polymer (FRP) composites is gaining increasing popularity in impact-resistant devices, automotives, biomedical devices and aircraft structures due to their high strength-to-weight ratios and their potential for impact energy absorption. Impact-induced high loading rates can result in significant changes of mechanical properties (e.g., elastic modulus and strength) before strain softening occurs and failure characteristics inside the strain localization zone (e.g., failure mechanisms and fracture energy) for fiber-reinforced polymer composites. In general, these phenomena are called the strain rate effects. The underlying mechanisms of the observed rate-dependent deformation and failure of composites take place among multiple length and time scales. The contributing mechanisms can be roughly classified as: the viscosity of composite constituents (polymer, fiber and interfaces), the rate-dependency of the fracture mechanisms, the inertia effects, the thermomechanical dissipation and the characteristic fracture time. Numerical models, including the viscosity type of constitutive models, rate-dependent cohesive zone models, enriched equation of motion and thermomechanical numerical models, are useful for a better understanding of these contributing factors of strain rate effects of FRP composites.

## 1. Introduction

Fiber-reinforced polymer (FRP) composites are increasingly used in impact-resistant devices, automotives, biomedical applications and aircraft structures due to their high strength-to-weight ratios and their potential for impact energy absorption [1,2,3,4,5,6,7,8,9,10]. FRP composites are often treated as softening materials, i.e., materials that show a reduction of the load-carrying capacity accompanied by increasing (localized) deformations after reaching the maximum load-carrying capacity [11,12]. Beyond the failure load, i.e., when the strain softening starts, gradual fracturing (strain localization prior to separation by cracking) occurs, which may manifest itself as various forms of damage such as matrix cracking, delamination, fiber breakage, etc. [13]. High strain rates generated by impact loading may cause significant changes in mechanical properties (e.g., elastic modulus and strength) before strain softening occurs and failure characteristics (e.g., failure mechanisms and energy dissipation) in the strain localization zone during the strain softening period [14]. In general, these phenomena are called the strain rate effects of FRP composites, and it can be divided into two categories: (1) the rate-dependent deformation of composites before strain softening occurs and (2) the rate-dependent failure process occurring in the fracture process zone where microcracks emerge, coalescence and develop into the specimen after strain softening starts. Understanding the strain rate effects is crucial for an accurate evaluation of the load-bearing capacities of composites and a better design of impact-resistance composite structures.

**Figure 1:**
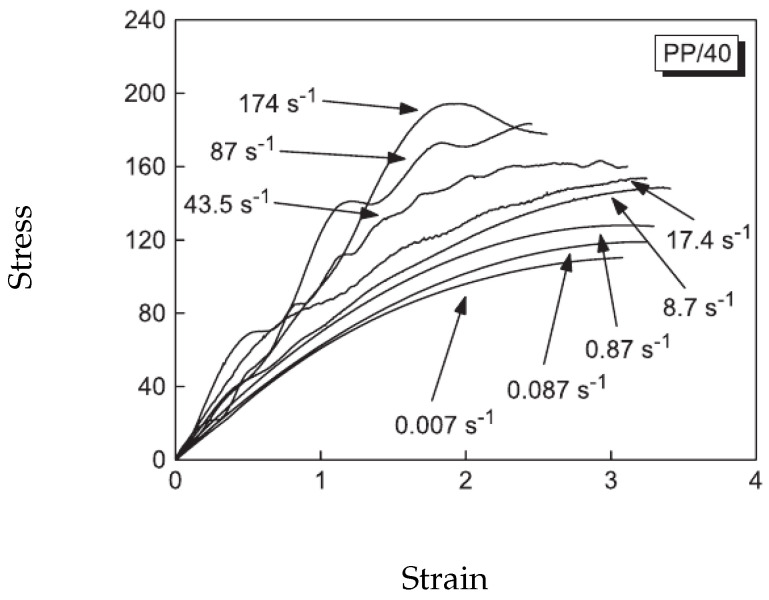
Stress-strain diagrams for PP with 40% glass fibers at different strain rates [15].

**Figure 2:**
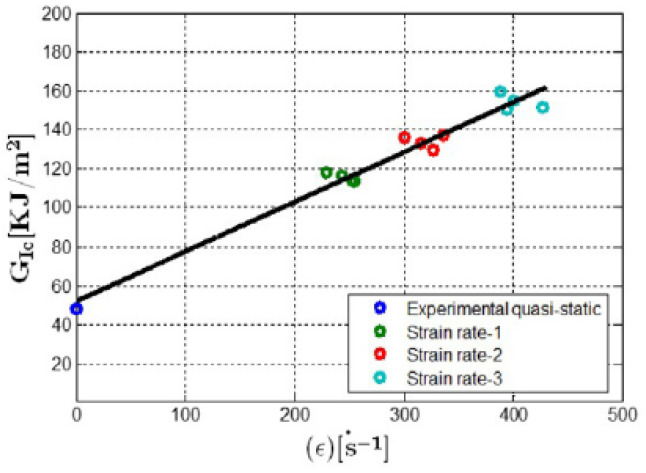
Mode-I fracture toughness versus strain rate of a carbon fiber reinforced composite [16].

**Figure 3:**
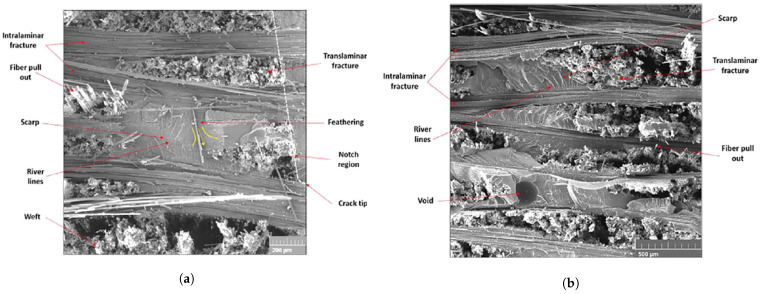
(**a**) The fracture surface of the specimen tested in the quasi-static regime with magnification of 150×. (**b**) Details on the transverse fibers of the specimen tested at strain rate of =245 s with magnification 110× [16].

As an example of the first category of the strain rate effects, Figure 1 depicts the stress–strain relation of a kind of glass-fiber-reinforced polypropylene (PP) composites for different strain rates ranging from 0.007 s${}^{-1}$ to 174 s${}^{-1}$ reported in Schoßig [15]. Remarkable differences in the stress–strain curves are observed for different strain rates, indicating that the elastic modulus and the strength can change with the loading rate. As an example of the second category, the investigation of Leite et al. [16] on a carbon/epoxy plain wave composite shows that the average fracture toughness of the studied composite increased from 45.88 to 77.50 MPa$\sqrt{m}$ from a static to dynamic regime with a strain rate of 410 s${}^{-1}$ (see Figure 2). The fracture surfaces of the tested specimen under quasi-static loading condition show that the translaminar fracture of the weft fibers combined with the intralaminar split of the resin along the fibers in the warp direction are the predominant failure modes (see Figure 3a). However, at strain rate of 410 s${}^{-1}$, the main failure mechanisms found were fiber pullout, translaminar and intralaminar failure modes (see Figure 3b).

The characterization of composite materials under different loading rates have been explored by various experimental systems, including the hydraulic servo machine, the Charpy pendulum, the drop weight, the Split-Hopkinson pressure bar and the plane impact test, covering a wide range of loading rates from static to shock wave loading [17,18]. It has been found that the mechanical behavior of FRP composites can show evident positive rate-dependency, rate-insensitive behavior or negative rate-dependency depending on the composite system (the type of fiber and matrix), the test rate range, loading type (tensile, compressive, shear, flexural etc.) and loading direction (longitudinal, transverse or off-axis) [15,19,20,21,22,23,24,25,26,27,28,29,30,31,31].

Early investigations on the characterization of the rate-dependent mechanical properties of composites before strain softening were carried out by Rotem and Lifshitz [32]. Rotem and Lifshitz [32] performed a series of tensile tests on unidirectional glass/epoxy composites with 60% volume fraction of fiber. Although results showed certain extent of scatter, it was discovered that tensile strength of the composite specimens under impact was three times higher than those of the static conditions. Longitudinal, transverse and shear loading tests, performed by Daniel and Liber [33], on unidrectional (UD) carbon/epoxy, glass/epoxy, graphite/epoxy and Kevlar/epoxy composites within a strain rate range of $10^{-4}$ to 27 s${}^{-1}$ showed different mechanical responses. Kevlar/epoxy composite showed a 20% increase for either tensile modulus or failure strength in the longitudinal direction, besides 40% and 60% in transverse and shear loading directions, respectively. However, the tensile modulus and failure strength of the carbon/epoxy, glass/epoxy, and graphite/epoxy composites were found be rate insensitive. Harding and Welsh [34] carried out dynamic tensile tests on UD graphite/epoxy in specimens oriented at $0^{\circ }$ and $45^{\circ }$ to the principal reinforcement directions. Unlike graphite/epoxy, which showed no evident mechanical response variances under imposed strain rate range of $10^{-4}$ to 1000 s${}^{-1}$, the dynamic modulus and strength of glass/epoxy were found to be 2–3 times of the static values for the $0^{\circ }$ and $45^{\circ }$ specimens. The source of the increment of failure strength for glass/epoxy was identified as the change in the failure mode. Gilat et al. [19] studied the tensile responses of carbon/epoxy IM7/977-2 system, with layups of $90^{\circ }$, $10^{\circ }$, $45^{\circ }$ and [$\pm 45^{\circ }{]}_{s}$ under a strain rate range of $10^{-5}$ to 600 s${}^{-1}$. The results showed that all the specimens, irrespective of layups, demonstrated higher value of stiffness and maximum stress with increasing strain rates. Besides, the [$\pm 45^{\circ }{]}_{s}$ specimens were much more sensitive to strain rate compared with the other composites, which meant that the rate dependency of the composites were dominated by the resin behaviors. Ochola et al. [22] tested both carbon fiber reinforced polymer (CFRP) and glass fiber reinforced polymer (GFRP) with a single laminate configuration, viz. cross-ply (0${}^{\circ }$/90${}^{\circ }$) polymer matrix composites (PMC) at strain rates of 10${}^{-3}$ and 450 s${}^{-1}$. It was showed that the dynamic material strength for GFRP increased with increasing strain rates, but the strain to failure for both CFRP and GFRP decreased with increasing strain rate. Shokrieh and Omidi [23] carried out a series of compressive tests on a unidirectional glass fiber-reinforced polymeric composites by using a servo-hydraulic testing apparatus, ranging from a strain rate of 0.001 to 100 s${}^{-1}$. Both the compressive strength and modulus were found to increase with increasing strain rates, and the compressive strain to failure was approximately insensitive to strain rates. Massaq et al. [35] investigated the compressive mechanical response of PA6/glass composite in the transverse and longitudinal fibers directions at strain rates from 10${}^{-5}$ to 2500 s${}^{-1}$. Positive rate-sensitivity for the elastic modulus and failure stress were observed and the loading direction, viz. longitudinal or transversal, also influenced the magnitude of rate-dependency. Hosur et al. [25] investigated the compressive response of both a unidirectional and a crossply carbon/epoxy composite at three different strain rates of 82, 164 and 817 s${}^{-1}$ with a modified SHPB setup. It was observed that the strength and stiffness of the composite laminates (except for through the thickness samples) under high strain rate increased considerably compared with the corresponding static values. There was a strong influence of loading direction and layup configuration since the thickness loading exhibits the maximum peak stress followed by unidirectional laminate loaded along 0${}^{\circ }$, cross-ply loaded along 0${}^{\circ }$ and unidirectional laminate loaded along 90${}^{\circ }$ in the descending order. Zhang et al. [10] investigated unidirectional, cross-ply, quasi-isotropic and angle-ply carbon/polyamide composites at a number of different strain rates ranging from $2.2\times 10^{-4}$ to 2200 s${}^{-1}$. Different layup laminates exhibited different sensitivities. The elastic modulus difference of unidirection/cross-ply laminates decreased, but that of unidirection/quasi-isotropic or unidirection/angle-ply laminates increased with the increasing strain rate.

When strain softening starts, there exists a fracture process zone where microcracks emerge, coalescence and develop into the composite materials. Fracture energy and energy dissipation mechanism of composites are predominant for crack initiation, development and propagation. Therefore, the rate sensitivity of fracture toughness and the damage mechanism of composites under dynamic loadings are widely investigated. Smiley and Pipes [36] utilized the Double Cantilever Beam (DCB) tests to characterize the rate effects of Mode-I interlaminar fracture toughness in graphite/PEEK and graphite/epoxy composites by imposing crosshead speeds from 4.2 × $10^{-6}$ to 6.7 × $10^{-1}$ m/s. The initiation fracture toughness was selected as the measure of material toughness. The values of the fracture toughness of the graphite/PEEK composites were noted to be decreasing from 1.5 to 0.35 kJ/m${}^{2}$ over five decades of the opening rate, whilst the corresponding values of the graphite/epoxy material decreased from 0.18 to 0.04 kJ/m${}^{2}$ over four decades of the loading rate. Kusaka et al. [37] studied the rate dependence of Mode-I interlaminar fracture behavior in unidirectional carbon/epoxy composite laminates over a wide range of loading rates from 0.01 mm/min to 20 m/s. There was a distinct rate-sensitive transition region in the middle of the fracture toughness versus the loading rate diagram, where the fracture toughness values were decreased with the increasing rate. Despite this rate-sensitive region, the fracture toughness values were not influenced by the loading rate. Blackman et al. [38,39] tested the carbon/epoxy and carbon/PEEK composite laminate with the DCB specimens with a smallest test rate of 2 mm/min up to a largest test rate of 15 m/s. The crack propagation in the DCB test could be either stable or unstable (“stick/slip”) depending on the loading velocity and composite system. For the PEEK/carbon-fiber composite, there was no major decrease in the value of $G_{Ic}$ for either initiation or arrest with an increasing rate. In the case of the epoxy/carbon composites, the value of $G_{Ic}$ remained insensitive to the rate across the entire test-rate range, with the value being about 0.3 kJ/m${}^{2}$. Zabala et al. [40] investigated the loading rate effect on mode-I interlaminar behavior of unidirectional and woven composites with a double cantilever beam configuration under loading velocity ranging from 8.3 × $10^{-5}$ m/s to 0.19 m/s. The main $G_{Ic}$ reduction on the unidirectional composite (a 24% reduction) is given when the increasing testing loading rate changes from quasi-static to a dynamic (approximately 0.05 m/s). Leite et al. [16] carried out experimental studies on the mode-I intralaminar tensile fracture toughness of a carbon fiber-reinforced composite subjected to high strain rates. The experimental results showed that the intralaminar fracture toughness of the studied composite laminates are very sensitive to the strain rate effects, indicating a linear dependency of the fracture toughness $K_{Ic}$ and strain energy release rate $G_{Ic}$ on the strain rate. Liu et al. [41] utilized a novel dual electromagnetic Hopkinson bar apparatus to test a double cantilever beam specimen of a unidirectional carbon/epoxy laminate T700/MTM with velocities in the range of 10–30 m/s. Considering the time derivative of energy release rate as the strain rate, the Mode-I interlaminar crack initiation fracture toughness of T700/MTM showed a strongly positive rate sensitivity for this system under dynamic loading conditions. Smiley and Pipes [42] carried out an experimental study of the rate effects of the fracture toughness in Mode-II in the graphite/PEEK and graphite/epoxy composite laminates with End Notched Flexure tests. The studied crosshead speeds ranged from 4.2 × $10^{-6}$ to 9.2 × $10^{-2}$ m/s. The results showed that the fracture toughness values at the onset of critical crack growth for graphite/PEEK material decreased monotonically from 1.9 kJ/m${}^{2}$ to 0.40 kJ/m${}^{2}$. While for the graphite/epoxy composite specimens, these values decreased from an initial value of 0.46 kJ/m${}^{2}$ to a plateau around 0.06 kJ/m${}^{2}$. Guimard et al. [43] utilized a modified Cracked Lap Shear (CLS) mode-II test configuration to investigate the dynamic delamination of fiber-reinforced plastics composites up to 10 m/s loading velocity. The fracture energy is found to be increasing with crack speed. The Mode-III interlaminar fracture toughness, $G_{IIIC}$, of a fiber-reinforced thermoplastic and a fiber-reinforced thermosetting matrix was investigated by Pennas et al. [44] using the edge crack torsion (ECT) test geometry. The employed materials were a unidirectional glass fiber-reinforced polypropylene with a fiber volume fraction of 35% and a woven glass/epoxy with a fiber volume fraction of 45%. The interlaminar fracture toughness of both types remained nearly constant over the considered crosshead displacement rate range of 0.2 to 200 mm/min, indicating that the fracture toughness values were not rate-dependent. It could be found that the dynamic fracture toughness sometimes could have considerable variance with that of static fracture toughness, and the experimental test results do not always show good consistencies among different researchers. Actually, discrepancies on the influence of the strain rate on the fracture toughness could exist even for the same kind of composite system. For the same carbon/epoxy composites, Aliyu and Daniel [45] found that $G_{IC}$ increased by up to 20% over the three decades of the loading rate. On the other hand, Smiley and Pipes [36] found that $G_{IC}$ remain nearly constant over three decades of low loading rates and then decreased up to 70% over the next decade of the strain rate. For a more comprehensive review on this topic, please refer to [46,47].

There exists a number of explanations about sources of the rate-dependency. As pointed out by Camacho and Ortiz [48], rate-dependent numerical results originate from the underlying competing factors, including characteristic scales (length and time) of the cohesive model and inertia. However, Corigliano et al. [49] claimed that the above explanations could not justify the phenomenon under a slow crack speed, whereby inertial effects do not play a role, but polymeric matrix composites still show tangible rate effects. The rate effects should be attributed to the viscous behavior of these materials. Hauch and Marder [50] found that the appearance of branching traverse cracks and a subsurface damage zone results in the velocity-dependency of fracture energy of Homalite-100 and PMMA. Similar explanations are given in Zhou et al. [51] by a crack propagation experiment of a pre-strained PMMA strip. It was shown that the increase in fracture energy with larger crack velocity was accompanied by the transition from smooth fracture surfaces to rather coarse fracture surfaces induced by small branching cracks. Besides, there also exists several speculations addressing the discrepancies observed in the experimental results. Guimard et al. [43] pointed out that rate effects for composites were not the same for crack velocities below the Rayleigh wave speed compared with intersonic or even supersonic propagations. It was mainly due to the complexity of the loading device, which led to strong interactions with the specimen itself. Another interesting explanation given by Landis et al. [52] about a polymer was that there existed a competition between the hardening rate of the bulk solid, which enhanced crack growth and the rate strengthening of the fracture process zone, which resisted propagation. Therefore, the results for material parameters characteristic of polymers show that the toughness of the material can either increase or decrease with increasing crack velocity. This kind of theory may also apply in FRP composites.

In this review, the multiscale mechanisms of the strain-rate effects in FRP composites and the corresponding useful numerical tools used to understand these mechanisms are discussed in detail. It is organized as follows: In Section 2, the underlying multiscale mechanisms of the observed strain rate effects in composites are discussed. This section explains the contributing factors across different length and time scales. In Section 3, numerical models developed to describe the mechanisms of the strain rate effects are presented. The final section gives a short summary about this review.

## 2. Rate-Dependent Deformation and Failure

It is identified that the underlying mechanisms of the observed rate-dependent deformation and failure of composites take place among multiple length and time scales. The contributing mechanisms can be roughly classified as:

- viscosity of composite constituents (polymer, fiber and interfaces) [53,54];
- rate-dependency of the fracture mechanism as it is constituted by the different failure processes (e.g., fiber failure with fiber pullout, matrix damage and fiber–matrix interface failure) occurring at microscale level under different loading rates [14,16,55];
- inertia effects characterized as inertia resistance against rapid deformation, damage formation and crack propagation. Due to material heterogeneity, micro-inertia effects also arise as a result of wave reflection occurring at the interfaces between the constituents, which can result in complex spatiotemporal scenarios of damage and failure evolution, initiated at multiple spots [56,57,58,59];
- thermomechanical dissipation as a transition from isothermal to adiabatic deformation and failure process is expected for increasing loading rate [60,61];
- the characteristic fracture time, as there is a threshold time (characteristic fracture time) required to activate cracks [48].

### 2.1. Rate-Dependent Fracture

**Figure 4:**
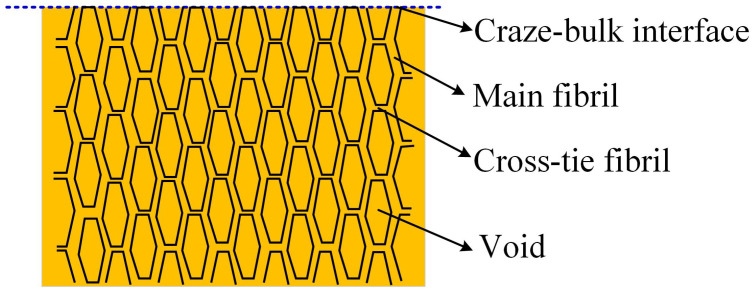
Schematic illustration of the craze structure.

A classical example of rate-dependent fracture process zone is the crazing process in glassy polymers [62]. As it was pointed out by Knauss [63], craze growth was clearly a time-dependent process. The structure of the craze was revealed as fibrils (main and cross-tie fibrils) separated by the voids (see Figure 4). Direct measurements of craze shapes for several glassy polymers, including polystyrene, poly(vinyl chloride) and polycarbonate, had confirmed the similarity to the plastic zone model proposed by Dugdale for metals. Ward et al. [64] pointed out that crazing occurs at a crack tip or in a solid section with a very appreciable increase in volume, which could be correlated to the hydrostatic tensile stress in craze initiation and growth. The theory for crazing growth has been well developed now. Based on the meniscus instability criterion proposed by Argon and Salama [65] for craze propagation, Kramer [66] showed how resistance to craze propagation increases with entanglement density and was therefore dependent on chain length and molecular characteristics. However, the craze initiation is still poorly understood, with simply treating crazing as a distinctive type of yielding dictated by standard yield criteria [67]. Crazes are usually initiated from microscopic surface flaws or embedded dust particles. Knauss [63] claimed that the unstable motion of the meniscus, which shaped a primordial craze, could require the possibility of viscous flow provided in the bulk polymer at points of high stress concentrations. The craze growth, thickening and failure processes are only qualitatively understood but are clearly time-dependent.

**Figure 5:**
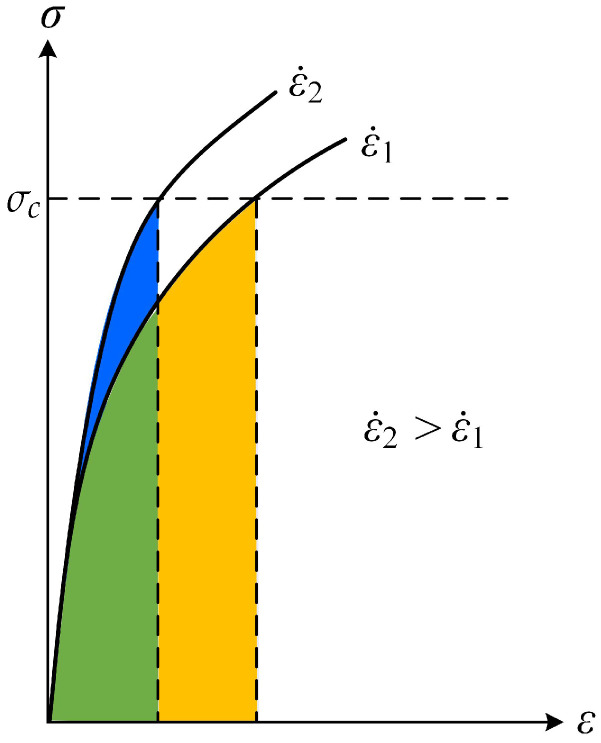
Qualitative explanation of strain rate dependence of a brittle fracture.

The decrease in fracture energy with increasing loading rates is possibly due to the strain rate dependence of a brittle fracture type, as explained in [68]. As it is described in Figure 5, a brittle fracture can be assumed to be produced, as a first approximation, when the stress at the crack tip reaches a critical level at which separation occurs, $\sigma_{c}$. The flow stress of materials is strain rate-dependent. If we equate, to a first approximation, this deformation energy to the energy release rate of a material, we will have a greater area at the lower strain rates. From a material point of view, the mechanism for the initiation of crack and propagation is different. When the crack tip is in stationary, the plastic flow can develop more freely, which screens the crack tip from the applied loading (see [69]). The stress should be elevated to enough magnitude to cause the crack tip to move. While during the crack propagation, the fracture is more brittle because the material does not have enough time to accumulate plastic deformation. Therefore, a more brittle failure type during crack propagation requires a smaller energy release rate than a more ductile failure type at initiation. For instance, for quasi-static tests, delamination is often dominated by fiber–matrix interface failure, while resin rich brittle fracture zones have been found more dominant in dynamic tests [46]. The extent of plastic deformation may decrease with an increased loading rate, which represents a ductile-to-brittle transition in the fracture process zone. That is why the crack initiation toughness $G_{i}$ can be larger than the crack propagation toughness [70]. Experimental evidence also shows that it is possible that the crack initiation toughness becomes larger than the propagation value. In Blackman et al. [39], the dynamic energy release rate is calculated using the modified beam theory for epoxy/carbon fiber composites subjected to DCB tests with a loading rate of 0.65 m/s. It is found that, at initiation (*a* = 35 mm), the energy release *G* is the maximum value, 0.19 KJ/m${}^{2}$, while the value of *G* during crack propagation (a>35 mm) is smaller.

**Figure 6:**
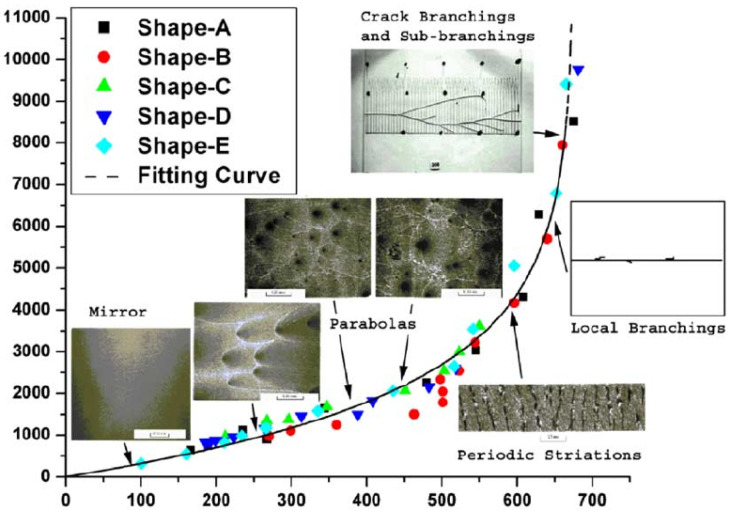
Fracture energy $G_{c}$ and the fracture characteristics corresponding to different crack speed regions for a Polymethyl Methacrylate (PMMA) plate [51].

**Figure 7:**
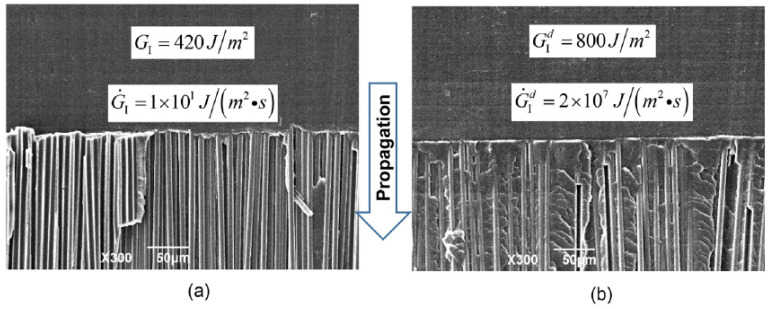
A typical scanning electronic microscopy image of DCB fracture surfaces at a magnification of 300 under (**a**) quasi-static and (**b**) dynamic loading [41].

**Figure 8:**
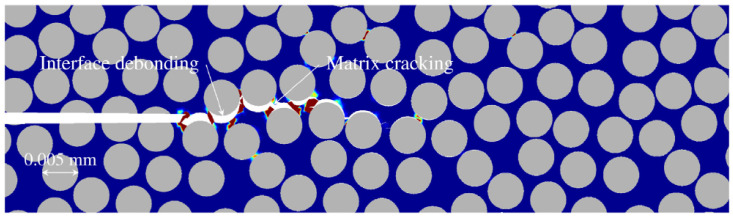
Microcracking in glass fiber-reinforced polymer composites [73].

**Figure 9:**
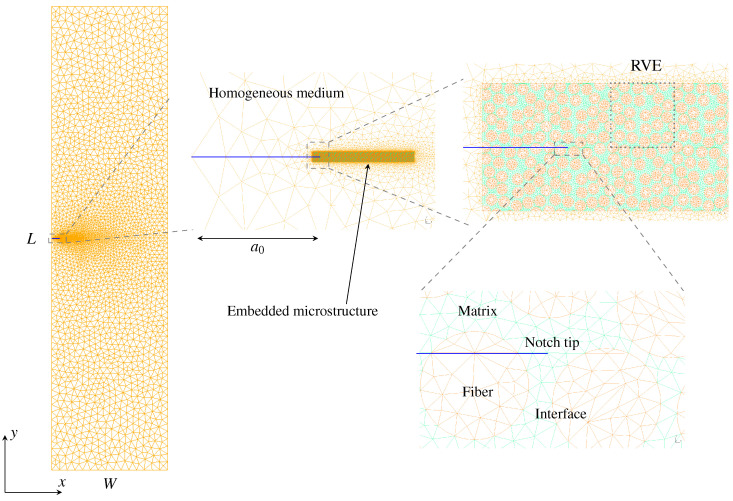
A finite element model of the SENT specimen. An initial notch is located on the left side of the specimen with an embedded microstructure represented by a number of repeating RVEs with stochastic fiber distributions. The dotted box on the top right shows the mesh of a RVE. Finer mesh is used for the embedded microstructure zone, and coarser mesh is used for the surrounding homogeneous medium [73].

**Figure 10:**
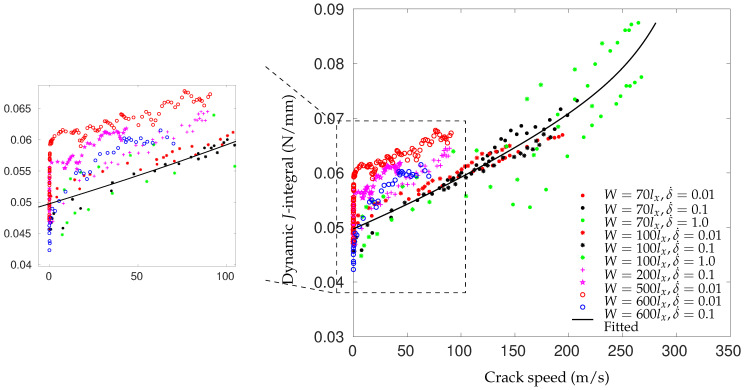
The dynamic *J*-integral for different crack speeds computed from the series of tests. A zoomed-in view of the lower crack speed range is shown on the left [73].

**Figure 11:**
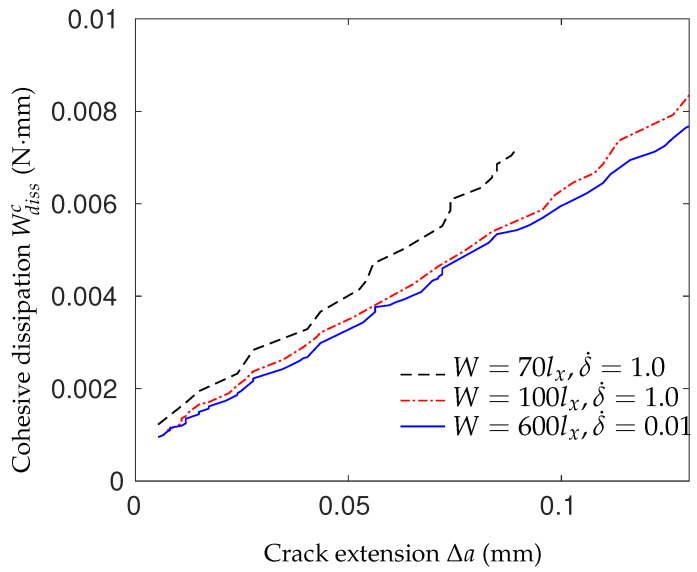
Cohesive dissipation for three cases: $W=70l_{x}$ and $\dot{\delta }=1.0$ m/s, $W=100l_{x}$ and $\dot{\delta }=1.0$ m/s and $W=600l_{x}$ and $\dot{\delta }=0.01$ m/s [73].

However, the fracture energy can increase with the loading rate as well. Experimental studies show that for a pure polymeric matrix, corresponding to different levels of propagation velocity, the crack surface roughness is observed to demonstrate different features since materials in the fracture process zone might experience high strain-rate plasticity, microcrack nucleation, thermomechanical interaction and other complex deformation/failure mechanisms [51,71]. As shown in Figure 6, with the increase in crack speed, the crack surface appears first to be almost flat (mirror regime), after which a rougher surface with conic marks appears (mist regime) and, finally, (micro)branching takes place (hackle regime). The increase in the apparent fracture toughness with a crack extension is usually described by a function of crack growth resistance vs. crack extension, i.e., the so-called R curve [72]. A microscopic examination of the delamination surfaces of unidirectional T700/MTM28-1 carbon/epoxy composites shows the difference between the dynamic delamination mechanism and the quasi-static delamination [41]. The smooth fracture surface of the quasi-static specimen suggests failure behavior dominated by fiber–matrix interface debonding (see Figure 7a). However, for dynamic delamination, extensive microbranching was observed in the matrix material in addition to the fiber–matrix interfacial fracture (see Figure 7b). As Guimard et al. [43] pointed out, for higher loading rate scenarios, a certain amount of energy is no longer used to speed-up the main crack in the same initial direction but to create new microscopic crack surfaces at specific non-zeros angles. Liu et al. [73] also observed microscopic cracks in a glass fiber-reinforced polymer composite under dynamic loading with an embedded cell numerical model of the single-edge-notched-tension (SENT) geometry with a width of W and a length of L (see Figure 8). In this study, a series of SENT tests was performed for different loading velocities and specimen sizes, while the dynamic energy release rate was evaluated using the dynamic *J*-integral. As it is shown in Figure 9, an initial notch of length $a_{0}$ along the x-direction was created on one edge of the specimen, and a symmetric displacement loading was applied on the top and bottom edge of the specimen with a prescribed velocity of $\dot{\delta }$. In the vicinity of the initial notch tip, a composite microstructure of 2 rows and 20 columns of repeating representative volume element (RVE) of length $l_{x}$ was embedded in a homogenized medium of the composite. The RVE had a stochastic distribution of 5 × 5 fibers with a fiber diameter of 5 μm and a fiber volume fraction of 60%. The fiber was modeled as a linear elastic material, and the polymer matrix was described by a viscoelastic-viscoplastic material model. Cracking was allowed to develop only inside the matrix and on the fiber–matrix interfaces in the embedded cell and were considered with the cohesive zone model. The maximum nominal strain rate investigated was 250/s. Figure 10 shows the dynamic *J*-integral value for different crack speeds extracted from the series of numerical tests. The differences in dynamic *J*-integral are numerical representations of the velocity-toughening effect that has been observed experimentally for quasi-brittle materials. Figure 11 shows a comparison of the dissipation of cohesive cracks for three cases, representing the lowest loading rate and the two highest loading rates. It is observed that the higher rate cases have significantly larger cohesive dissipation, pointing at more damage in the secondary microcracks.

### 2.2. Viscous Composites

**Figure 12:**
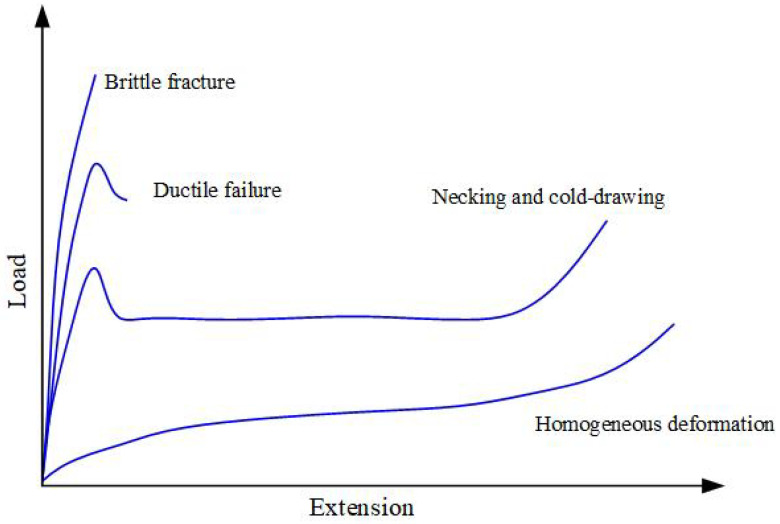
Load-extension curves for a typical polymer tested at four temperatures, showing different regions of mechanical behavior: brittle fracture; ductile failure; necking and cold-drawing and homogeneous deformation (quasi-rubber-like behavior).

The rate-dependency of the composites can be originated from the inherent individual phases, including polymer, fiber and interfaces. The rheology of the polymer matrix, through viscoelasticity or viscoplasticity, governs the non-linear and time-dependent behavior of the composites. Polymer is a big class of material, which could exhibit a large swathe of mechanical properties because of the enormous possibilities of their molecular and supra-molecular morphology [63]. According to Kinloch [74], polymers can be separated into three distinct groups: thermoplastics, rubbers (elastomer) and thermosets. In addition, thermoplastics can be separated into two subgroups: crystalline and non-crystalline (amorphous) thermoplastics. Thermoplastics are linear or branched polymers that melt upon heating. Rubbers are lightly cross-linked polymers that have elastomeric properties, and thermosets are rigid, highly cross-linked polymers that degrade rather than melt upon the application of heat. In general, polymers can show distinctive types of behavior depending on the exact working conditions, quite irrespective of their chemical nature and physical structure. Two predominant factors that influence the mechanical properties of polymers are temperature and strain rate. Typically, the load-elongation curve at a constant strain rate changes with increasing temperature, as shown schematically in Figure 12. At low temperatures, the load follows a nearly linear rise with increasing elongation up to the breaking point, leading to a brittle fracture manner. At higher temperatures, a yield point is observed, and the load falls before failure, sometimes with the appearance of a neck, i.e., ductile failure, but still at quite low strains (typically 10–20%). At still higher temperatures, under certain conditions, strain hardening occurs, the neck stabilizes and cold-drawing ensues. The extensions, in this case, are generally very large, even up to 1000%. Finally, at even higher temperatures, homogeneous deformation is observed, with a very large extension at the break.

**Figure 13:**
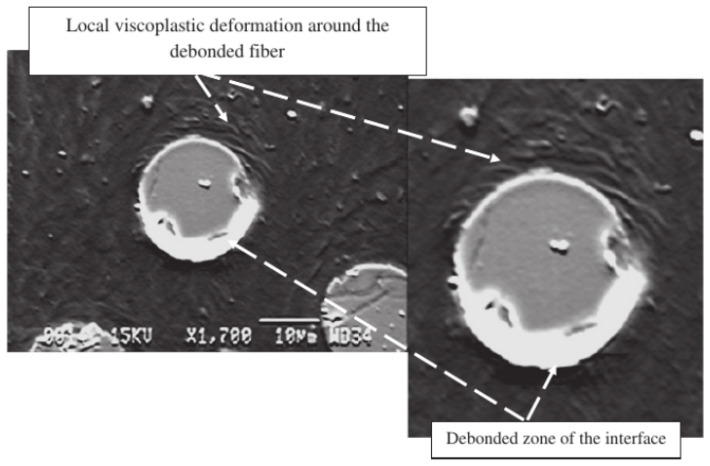
Matrix local viscoplastic deformation around a debonded fiber [53].

**Figure 14:**
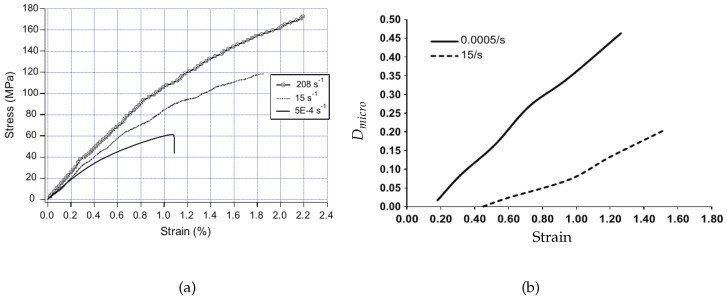
(**a**) Typical EPC matrix composite stress–strain tensile curves. The three characteristic stages can be shown for all strain rates; (**b**) Comparison between dynamic and quasi-static damage kinetic at the microscopic scale for all fiber orientations considered as an overall microscopic damage indicator [53].

The viscoelasticity of composites can be directly linked to the viscoelasticity of the polymer matrix, as demonstrated in Courtois et al. [75]. A temperature- and cure-dependent linearly viscoelastic model was derived by homogenization of the 3D interlock woven carbon/epoxy composite laminate. This model is then validated on experimental composite’s creep responses, showing that the temperature-dependent predictions agree well with experimental data in the composite’s linear domain below the glass transition temperature. Similarly, the viscoplasticity of composites also originates from the viscosity of the polymer matrix. It is shown in van der Sluis et al. [76] that composites with elastoviscoplastic matrix material and soft irregularly distributed, incompressible elastic inclusions can be described by Perzyna’s viscoplastic model. The comparison of the heterogeneous and homogeneous simulations reveals that the obtained global results match quite well for this non-linear problem. Moreover, an experimental study of a discontinuous glass fiber-reinforced ethylenepropylene copolymer (EPC) matrix composite reported in Fitoussi et al. [53] also shows that the viscosity observed at the macroscopic scale is controlled by the EPC matrix viscous rheology. This is also exemplified by the typical deformation pattern shown in Figure 13, in which the local viscoplastic matrix is developed around the fibers. In contrast to the quasi-static loading, no matrix micro-cracking was observed around the debonded interfaces. Furthermore, as reported in Figure 13, one can observe that the matrix is highly strained locally around the fibers. Therefore, the strained zone around the debonded interface dissipated the strain energy and consequently inhibited the interfacial crack propagation through the matrix. This resulted in the delayed damage threshold in dynamic loading compared with the quasistatic test (see Figure 14b). It should be noticed that the delayed damage threshold accounts for the high stress corresponding to the appearance of non-linearity observed on the macroscopic stress–strain curve in Figure 14a.

### 2.3. Inertia Effects

**Figure 15:**
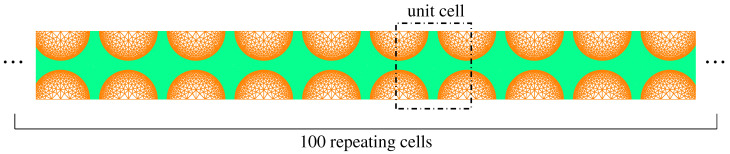
The DNS model mesh of a composite structure consisting of 100 repeating unit microstructure cell. There are two phases of materials, circular inclusions with a diameter of 0.5 mm and a surrounding matrix [78].

**Figure 16:**
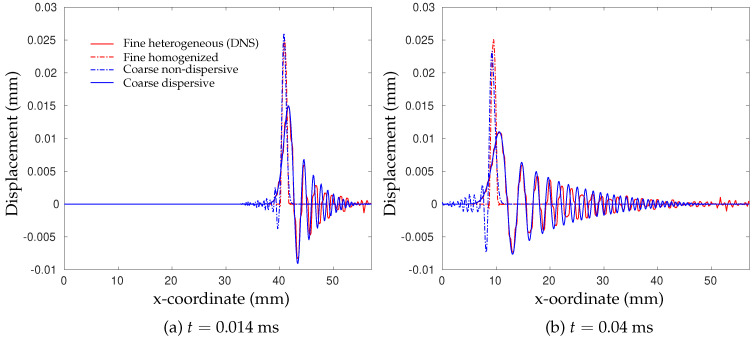
Plots of the displacement field at different time [78].

The inertia effect is recognized as a multi-scale problem, which should be examined at both the microscale and the mesoscale. At the microscale level, Fish et al. [77] stated that for high rates of loading and a long observation time, internal material interfaces in a heterogeneous material cause reflection and refraction of stress waves, giving rise to dispersion and attenuation of waves within the material microstructure. This phenomenon was further investigated by Liu et al. [78] using a dispersive multiscale numerical model. Elastic wave propagation in a two-dimensional composite microstructure subjected to an incoming sinusoidal wave was considered. The geometry consists of 100 repeating microstructures, and its microstructure has two phases of materials, circular inclusions and a surrounding matrix (see Figure 15). The top edge and bottom edge of the structure were fixed in a vertical direction, which, together with plane-strain conditions, mimics the state for materials in the middle region along the thickness direction for a plate impact test. Four numerical models were considered: the fine heterogeneous model (DNS), the fine model with homogenized properties, the coarse non-dispersive multiscale model and the coarse dispersive multiscale model. Among them, the DNS model kept the different properties of each phase, and therefore, the dispersion caused by material heterogeneity was naturally included. The other three models neglected the contribution of dispersion at the macro-scale or used coarser mesh. The horizontal displacement $u_{1}$ of the incoming wave at the right edge satisfies $u_{1}=A_{0}\sin\left(2\pi ft\right)H\left(\frac{1}{2f}-t\right)$ with $A_{0}=0.025$ mm and f=400 KHz with constrained vertical displacement $u_{2}=0$. The elastic properties of inclusion and matrix had a 100-times difference. The averaged horizontal displacement u(x) along the bar for two time instants is plotted in Figure 16. According to the reference DNS solution, the input sinusoidal wave does not maintain its profile during the propagation, and it breaks into several pulses with obvious magnitude decay. The profile also shows significant oscillations caused by reflections at material interfaces.

**Figure 17:**
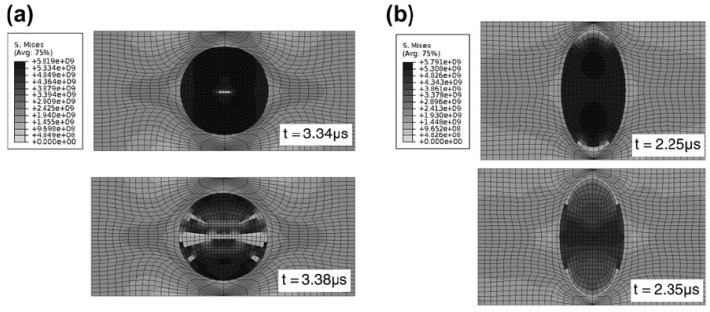
Contour plots of the von Mises stress at different times: (**a**) cracking in the middle of the fiber and (**b**) failure at the fiber–matrix interface [56].

As a heterogeneous material, the internal interfaces of the composites might influence the stress wave propagation inside the micro-structure and cause the local stress distribution and damage mechanism in dynamic loading cases to be remarkably different from that of static loading cases. With micro-mechanical analysis, Chen and Ghosh [56] found that the microstructural morphology of the representative volume element (RVE) consisted of SiC fiber and Al7075-T6 matrix led to different damage mechanisms, energy absorption and dissipation characteristics (see Figure 17). Figure 17a depicts the damage and failure process in the RVE with a unidirectional cylindrical fiber under compressive loading. It indicates that fiber cracking in the middle of the fiber is the predominant damage mode. Cracking begins at a time of 3.34 μs. For another microstructure containing elliptical fibers, numerical simulations in Figure 17b shows that the failure begins at the interface at a time of 2.25 μs. The difference of the failure mechanisms is due to the stress wave profile in the two composite microstructure.

**Figure 18:**
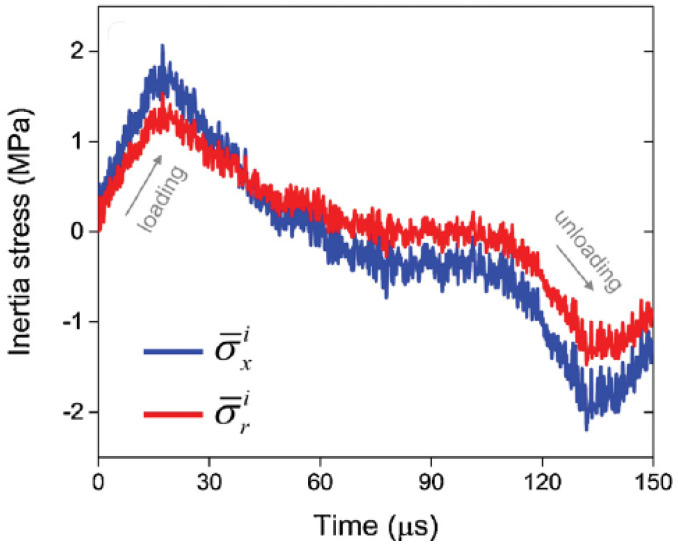
Axialand radial inertia stresses with respect to the deformation time. Strain rate data were extracted from a controlled impact test performed on a polymer foam in a Split-Hopkinson pressure bar. Compressive stress is positive [79].

At the mesoscale level, inertia effects can arise either from rapidly applied loading on a cracked solid or from rapid crack propagation and are characterized as inertia resistance against rapid deformation, damage formation and crack propagation [69]. For instance, radial inertia is one critical issue in dynamic characterization of very soft materials with a Kolsky compression bar. When subjected to axial compressive loading in a Kolsky compression bar test, the specimen becomes radially expanded due to Poisson’s effect. The lateral acceleration becomes a constraint such that an apparently higher force is needed to compress the specimen. As a consequence, the additional radial-inertia-induced axial stress contributes to the strain rate effect [79]. In this study, a polymer foam was dynamically characterized in compression with the Kolsky compression bar. The radial inertia stresses of the polymer foam material is shown in Figure 18, together with the axial inertia stress. As seen in Figure 18, radial inertia can have a remarkable influence, and the accurate identification of elastic parameters will not be possible unless the inertia stresses are accurately measured.

### 2.4. Thermomechanical Effects

The influence of the strain rate on the polymer’s mechanical response can be understood through the correlation between the strain rate, the temperature and the time scale. The correlation between the temperature and time scale is known as the time-temperature equivalence in the polymer. However, the connection between the strain rate and temperature is built on the fact that the strain rate could change the viscoelastic transition (α, β, etc.) temperature. Mulliken and Boyce [80] studied the rate-dependent elastic-plastic deformation of two amorphous polymers-polycarbonate (PC) and PMMA subjected to uniaxial tension and compression at strain rates ranging from $10^{-4}$ to 10${}^{4}$ s${}^{-1}$. They claimed that there existed a considerable transition in the nature of the rate dependency of the material yield behavior as well as the viscoelasticity of a variety of amorphous polymers. The transition process is closely related to the primary (glass) α-transition and a secondary β-transition. The (glass)-transition for both PC and PMMA is triggered by restricted rotations and translations of the polymer main chains. However, the β-transition is associated with the molecular mobility of main-chain phenyl groups and the molecular mobility of the ester side groups with respect to the main chain for PC and PMMA, respectively. The transition temperature for both the α and β process is strain-rate-dependent. When the strain rate increases, the β-transition temperature also increases, which could lead to a significant contribution of the β-process. The same principle applies to the α-process.

**Figure 19:**
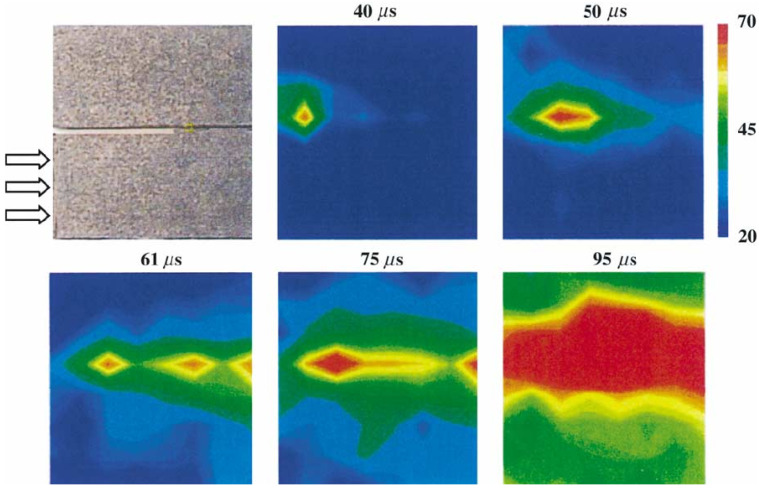
Experimental observations: high-speed infrared images of hot spot formation due to contact behind an intersonically moving shear crack. The top-left image shows the size of the exposed area. The following images show contours of a constant temperature at different time steps [81].

At high velocity loading conditions, such as impact, the process is nearly adiabatic, the energy, which is in excess of the energy required for the creation of new crack surfaces, can be dissipated as heat. This could result in a substantial increase in the local temperature near the crack tip region. Yu et al. [81] investigated the impact of a unidirectional graphite–epoxy composites with a projectile ranging from 10 to 57 m/s. Infrared thermal images showed that local hot spots were formed along the fracture surfaces of an intersonic shear-dominated crack (see Figure 19). In the tensile loading, the temperature of a carbon–epoxy composite can increase as much as 100 ${}^{\circ }$C (for a strain of 1500 s${}^{-1}$) [60]. This internal heat generation process can significantly influence the material’s mechanical response [61]. Mechanical properties of polymers change dramatically with temperature, going from glass-like brittle behavior at low temperatures to a rubber-like behavior at high temperatures. This behavior can be understood in terms of the structure of glassy materials, which are formed typically by substances containing long chains, networks of linked atoms or those that possess a complex molecular structure. Furthermore, a localized increase in temperature in the crack-tip region may affect the energy release rate of the crack and, consequently, the crack propagation speed [82].

**Figure 20:**
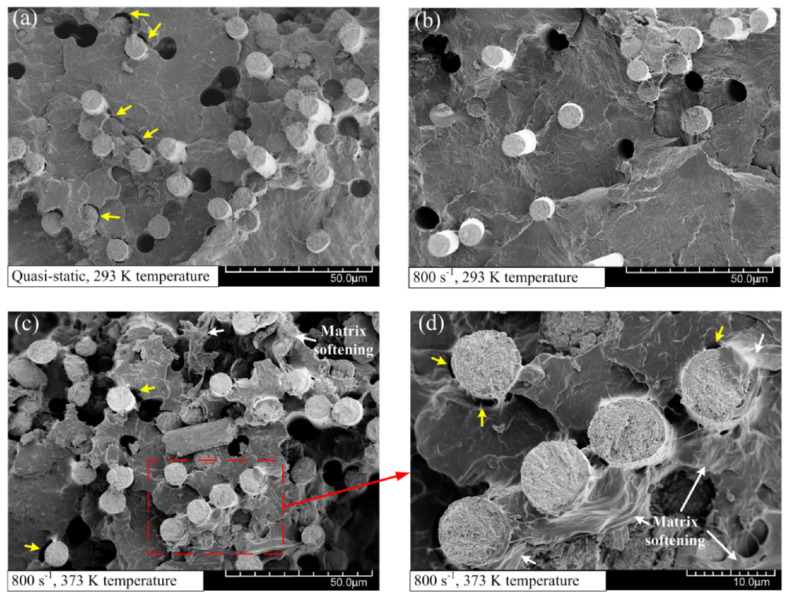
SEM micrographs of the fracture surface in layer at (**a**) quasi-static and 293 K temperature; (**b**) 800 s and 293 K temperature; (**c**) 800 and 373 K temperature and (**d**) its local enlargement view [10].

As an example, in [10], the failure characteristics of carbon fiber-reinforced polyamide 6 laminates for different strain rates and temperatures are presented. Figure 20 shows the micro morphologies of the fracture surface in the 0${}^{\circ }$ layer under different loading conditions. In Figure 20a, the PA6 matrix and longitudinal carbon fibers exhibit a brittle fracture under the quasi-static tension condition and a temperature of 293 K. Both fiber pullout and the fiber–matrix interface cracking are observed. As the strain rate increases, the river-like pattern and brittle matrix cracking become more obvious, as shown in Figure 20b. While both the temperature and strain rate increase, the failure morphologies demonstrate more breakage and complicated failure mechanisms, including fiber pull-out, fiber breakage, fiber–matrix debonding and matrix fracture (see Figure 20c,d). Furthermore, the matrix softening and subsequent plastic deformation marked by the white arrows may be attributed to the temperature raise. The matrix softening, especially in the vicinity of the fiber–matrix interface, accelerated the separation of the fibers and matrix. As a result, the tensile modulus and strength of the composites decreased significantly under a high-temperature environment.

### 2.5. Characteristic Fracture Time

**Figure 21:**
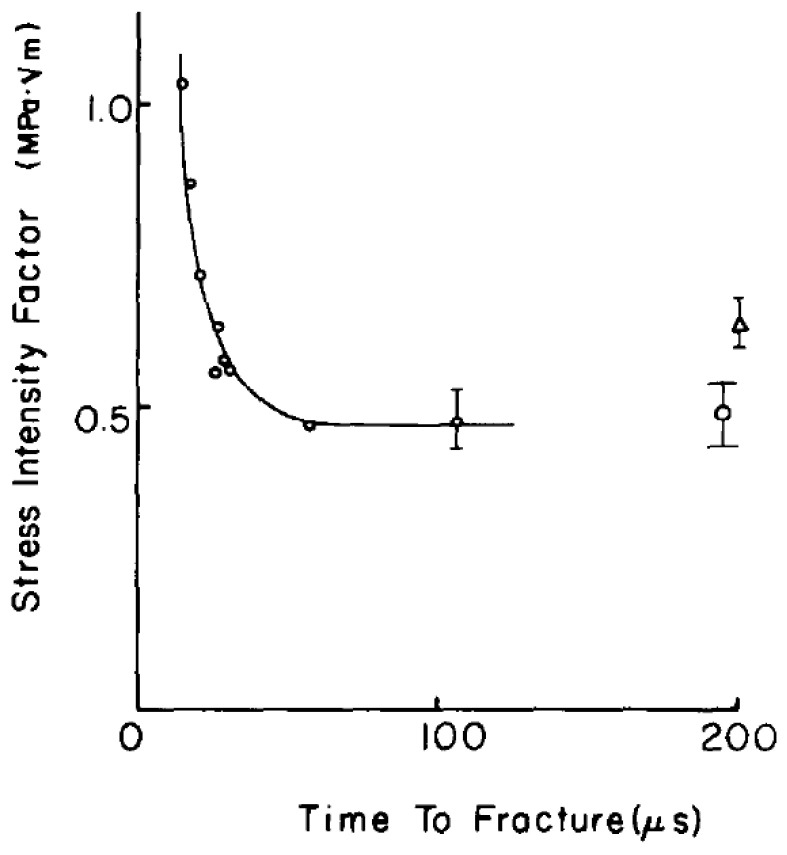
The stress intensity factor at initiation as a function of the time at initiation [83].

Meyers [68] pointed out that there is a threshold time (characteristic fracture time) required to activate cracks. When the duration of the applied pulse is less than the threshold time, the crack does not grow. This threshold time decreases as the stress is raised, which is confirmed with the Homalite-100 (a polyester resin) material by Knauss and Ravi-Chandar [83]. The result of this experimental scheme is shown in Figure 21, where the stress intensity factor at initiation is plotted against the time at which the crack was initiated.

**Figure 22:**
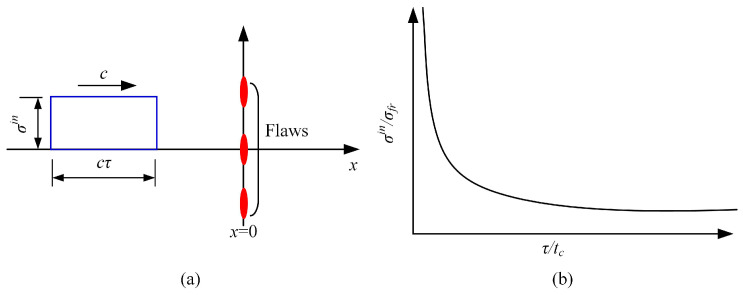
(**a**) The configuration for determining fracture time dependence. (**b**) time dependence of the incident stress $\sigma^{in}$ to cause fracture.

Shockey and co-workers [84,85] obtained similar results for AISI 4340 steel and 6061-651 aluminium alloy. With the framework of cohesive model, Camacho and Ortiz [48] investigated the problem by considering a spall plane with a collinear array of flaws at x=0, which was reached at t=0 by an incident square pulse of a magnitude of $\sigma^{in}$ and a duration of τ (see Figure 22a). When the incident wave impinged upon the spall plane, the required pulse duration time for spall to occur could be calculated using the wave equations and the assumed cohesive law. It was shown in Figure 22b that as the pulse duration decreased, the pulse amplitude should increase to cause spall to occur. This effect resulted in an apparent spall strength enhancement at high rates of loading.

## 3. Numerical Models for Strain Rate Effects

Numerical models can be used to investigate the contribution of the underlying mechanisms of strain rate effects of FRP composites since it is easier to isolate the contribution of different mechanisms with numerical models. In this section, numerical models for studying the mechanisms in Section 2 are reviewed. This includes a rate-dependent cohesive zone model, viscocity type of constitutive models, enriched equation of motion for inertia and micro-inertia effects, thermomechanical numerical model and numerical skills for considering the characteristic fracture time.

### 3.1. Rate-Dependent Cohesive Law

A popular strategy to deal with delamination in composites is to insert interface elements between laminae with an appropriate cohesive law, assuming localized energy dissipation between neighbouring plies. However, rate-independent cohesive interface elements are not able to capture the main features of the dynamic delamination process of a composite. This indicates that rate-dependency should be introduced into numerical models to account for the rate effects under dynamic loading scenarios. The overall rate dependence could stem from the rate-dependent properties of the bulk material, the cohesive zone or both. For the last case, it is still not clear how the coexisting rate-dependency between the bulk material and cohesive zone could interact with each other. Different modeling strategies have been reported based on the assumption made on the origin of the rate effects. In general, four classes of models have been proposed in the literature to incorporate rate effects into a cohesive interface for numerical modeling of the delamination of composites. They are recognized as the dynamic increase factor (DIF) model, the viscoplastic model, the damage-delay model and the viscoelastic model. The first class of models usually assumes that characteristic parameters of the chosen cohesive law (cohesive strength, fracture energy, etc.) are functions of the separation rate or crack velocity of the cohesive zone. In the wake of rate effects, these parameters are no longer constant during crack propagation. We call this class of models dynamic increase factor (DIF) models in this review for ease of reference. The seminal work of the dynamic increase factor model was accomplished by Glennie [86], where the steady-state crack propagation in metal with small-scale yielding was investigated, and the dynamic yield strength in the plastic zone was taken as dependent on the strain rate. The correlation between the dynamic yield strength and strain rate was assumed as follows:

(1) $$Y=Y_{0}+F\dot{\varepsilon }$$

where *Y* is the dynamic yield stress; $Y_{0}$ is the static yield stress; *F* is viscous factor; $\dot{\varepsilon }$ is the plastic strain rate. In the research work of Fager et al. [87] and Langer and Lobkovsky [88], Glennie’s model was reformulated by introducing damage effects into rate-independent strength parameters to alleviate the stress singularity that occurred at the tip of the cohesive zone. Kubair et al. [89] performed an analytical and numerical analysis on a semi-infinite steady-state crack propagation under mode III with Glennie’s model.

To model the rate-dependent performance of a crash-optimized adhesives, May et al. [90] developed a rate-dependent cohesive zone model in which the maximum stress and fracture energy of both mode I and mode II loading were relying on the strain rate of the cohesive elements. A number of tests, including static and dynamic butt joint tests, compressive double lap shear test, TDCB tests and TENF tests, were used to calibrate parameters of the introduced model. A series of impaction tests of a T-joint specimen were carried out as well as numerical simulations. A comparison of the impactor force with impactor displacement of the test results with numerical simulation results showed that the rate-dependent formulation could capture the major features of the test results well. This model is utilized in Liu et al. [70] for the study of dynamic delamination of fiber/PEEK composites in a number of DCB tests. Rate dependency was introduced for both the cohesive strength and the fracture energy of independent modes with a Johnson–Cook law similar to May [90]. For pure mode-I or mode-II, the following relation was introduced,

(2) $$\sigma_{i}\left(\dot{\Delta }\right)=\left\{\begin{array}{cc} \sigma_{i}^{0}\left(1+c_{i}\ln\left(\frac{{\dot{\Delta }}_{i}}{{\dot{\Delta }}_{i}^{\mathrm{ref}}}\right)\right), & {\dot{\Delta }}_{i}\geq {\dot{\Delta }}_{i}^{\mathrm{ref}}\\ \sigma_{i}^{0}, & {\dot{\Delta }}_{i}<{\dot{\Delta }}_{i}^{\mathrm{ref}} \end{array}\right.,\;i=1,2$$

here, $\sigma_{i}$ is the rate-dependent cohesive strength; $\sigma_{i}^{0}$ is the quasi-static cohesive strength; $c_{i}$ is a rate-sensitivity constant; ${\dot{\Delta }}_{i}$ is the displacement jump rate; ${\dot{\Delta }}_{i}^{\mathrm{ref}}$ is a reference displacement jump rate. The rate dependency of the fracture toughness is assumed to be

(3) $$G_{Ic}\left(\dot{\Delta }\right)=\left\{\begin{array}{cc} G_{Ic}^{0}, & {\dot{\Delta }}_{1}<{\dot{\Delta }}_{1}^{\mathrm{ref}}\\ G_{Ic}^{0}\left(1+m_{1}\ln\left(\frac{{\dot{\Delta }}_{1}}{{\dot{\Delta }}_{1}^{\mathrm{ref}}}\right)\right), & {\dot{\Delta }}_{1}^{\mathrm{ref}}\leq {\dot{\Delta }}_{1}\leq {\dot{\Delta }}_{1}^{\inf}\\ G_{Ic}^{\inf}, & {\dot{\Delta }}_{1}>{\dot{\Delta }}_{1}^{\inf} \end{array}\right.$$

(4) $$G_{IIc}\left(\dot{\Delta }\right)=\left\{\begin{array}{cc} G_{IIc}^{0}, & {\dot{\Delta }}_{2}<{\dot{\Delta }}_{2}^{\mathrm{ref}}\\ G_{IIc}^{0}\left(1+m_{2}\ln\left(\frac{{\dot{\Delta }}_{2}}{{\dot{\Delta }}_{2}^{\mathrm{ref}}}\right)\right), & {\dot{\Delta }}_{2}^{\mathrm{ref}}\leq {\dot{\Delta }}_{2}\leq {\dot{\Delta }}_{2}^{\inf}\\ G_{IIc}^{\inf}, & {\dot{\Delta }}_{2}>{\dot{\Delta }}_{2}^{\inf} \end{array}\right.$$

**Figure 23:**
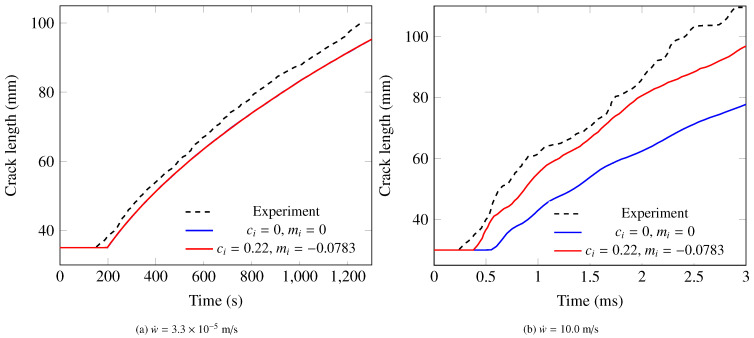
A comparison between experimental and cohesive zone modeling results for the test rate of (**a**) $\dot{w}=3.3\times 10^{-5}$ m/s; (**b**) $\dot{w}=10.0$ m/s [70].

The numerical models were used to simulate a series of DCB tests of a unidirectional PEEK/carbon composite laminate with different test rates from $3.3\times 10^{-5}$ to 10.0 m/s, as reported in [38,39]. Both the rate-independent (i.e., $c_{i}=m_{i}=0$) and rate-dependent cohesive laws were considered for each analysis. It can be seen from Figure 23a that the rate-independent model and rate-dependent model produce almost the same time vs. crack length curve for the lowest loading rate. They both match reasonably well with the measured curve. For the test rate of 10.0 m/s (Figure 23b), the time vs. crack length curve obtained with the rate-dependent model still matches reasonably well with the measurement in terms of crack speed, while the rate-independent model significantly underpredicts the crack speed. It is noted that the influence of the properties of matrix and fiber–matrix interface is considered by the values of the rate-dependent parameters in Equations (2)–(4).

As examples of the second class of methods, i.e., the viscoplastic cohesive zone model, a reference is made to Morin et al. [91], who used a non-zero thickness cohesive element with a coupled damage-viscoplasticity cohesive law to investigate the crushing of a bonded thin-walled closed-hat-section profile. The increase in the strain rate in the cohesive element would improve the yield stress of the cohesive material.

(5) $$\sigma_{y}=\sigma_{yo}\left(1+b\log\frac{\dot{\bar{\varepsilon }}}{{\dot{\varepsilon }}_{o}}\right)$$

Here, $\sigma_{y}$ is the yield stress; $\dot{\bar{\varepsilon }}$ is an equivalent strain, $\sigma_{yo}$; *b* and ${\dot{\varepsilon }}_{o}$ are three reference values, which are identified by relevant tests.

Corigliano and Ricci [92] presented two rate-dependent interface models: one is a viscoplasticity model in which the variation of viscoplastic displacement discontinuities follows a Perzyna-type evolution law, and another is an anisotropic time-dependent elastic-damage model whereby the rate of damage depends on a potential function. Analytical solutions of these two interface models under pure modes revealed that they both showed a monotonic trend for increasing the imposed displacement discontinuity rate. Comparisons of the numerical and experimental results of DCB and ENF tests showed the potentialities of the proposed formulation. Analogous rate-dependency description methods could refer to Lu and Xu [93], who presented a coupled damage-viscoplasticity cohesive zone model in which a Perzyna-type viscoplasticity model was utilized, and isotropic hardening of the interface material was also considered. Another related work was reported by Moreo et al. [94], who developed a coupled damage–viscoplasticity interface model for the simulation of fatigue failure of the cement–bone interface.

To study the mechanical behavior of bonded joints under mix-mode loading, Giambanco and Scimemi [95] introduced a viscoplasicity model derived from a standard thermodynamic consistent approach with Helmholtz free energy density for unit interface surface defined as

(6) $$\Psi \left({\left[u\right]}^{e},\xi ,\xi^{v}\right)=\frac{1}{2}{\left({\left[u\right]}^{e}\right)}^{T}E{\left[u\right]}^{e}+\frac{1}{2}H_{\infty }\xi^{2}+\frac{1}{2}H_{v}{\left(\xi -\xi^{v}\right)}^{2}$$

where the meaning of each parameter was illustrated in the article. The evolution of the viscoplastic discontinuity and internal variables are governed by two dissipation potentials of the Perzyna kind. The rate dependency of the interface material is captured by a Maxwell-type relation between a thermodynamic force and its conjugate internal variable. With well-calibrated material parameters, numerical simulations of the DCB test with different loading speeds are carried out and satisfactory agreements are achieved between the numerical results and the corresponding experiment test results of DCB specimens.

Matzenmiller et al. [96] introduced two modeling methodologies to study the delamination of composites and the failure of adhesive bonds. The first one was a damage model with two different formulations addressing single-mode traction separation and interactions among different modes, either bilinear traction-separation laws for a single-fracture mode along with a common mixed-mode interaction model or a Tvergaard and Hutchinson [97] model considering both single and mixed-mode behaviors. A Johnson–Cook model [98] was adopted to scale the cohesive strength parameters so that rate effects could be taken into consideration. The second one was an elastic-plastic model, which made use of Coulomb friction yield surface with tension-cutoff and considered initial yield stresses depending on the effective displacement discontinuity.

The third class of models are damage-delay models, which is proposed by Allix et al. [99]. The major features of this kind of rate-dependent cohesive zone mode are: firstly, damage evolution due to variations of forces is not instantaneous, and secondly, a maximum damage rate exists. Allix et al. [99] used the damage delay model to model a dynamic uniaxial tension of a perforated laminate [$\pm 25^{\circ }$] and showed the capability of such models to reproduce the magnitudes of the damage mechanisms in the plies and interfaces during the whole fracture process.

Guimard [43] adopted the damage delay model to simulate the delamination of composite fiber-reinforced plastics subjected to a modified Cracked Lap Shear mode-II test configuration. It was shown that the damage delay model could introduce a maximum crack velocity and created a correlation between the critical energy release rate and crack speed. The numerical results also showed that rate effects have to be considered in order to have a better match with experimental data.

**Figure 24:**
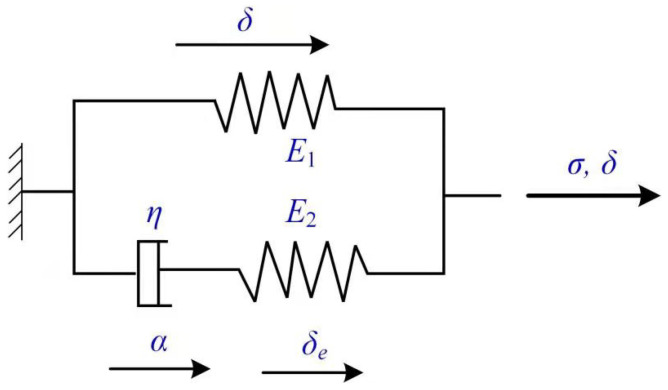
The viscoelastic model by Musto and Alfano [100].

The fourth category of models are viscoelasticity cohesive laws. Musto and Alfano [100] formulated a coupled damage-viscoelastic cohesive zone model following a thermodynamically consistent framework in which the rate-dependency of the overall fracture energy was due to the viscous dissipation of the interface. The rheological representation of the interface is shown in Figure 24. A good match between the numerical simulation and experiment results of the DCB specimen justified the validation of the proposed method for two logarithmic decades of loading rates. The use of a wider relaxation spectrum or of a more complex, possibly non-linear, viscoelastic law might be needed to cover wider displacement rate range.

**Figure 25:**
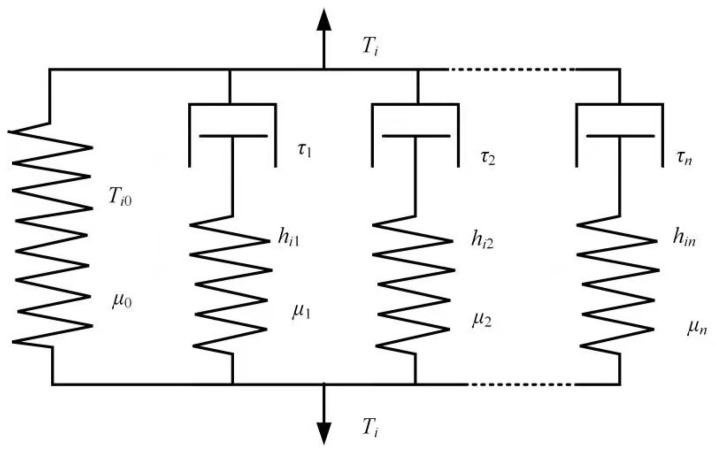
The viscoelastic model by Geibler and Kaliske.

Geißler and Kaliske [101] introduced two kinds of rate-dependent cohesive material formulations. The first one only considered the traction as a rate-dependent quantity and computed the traction by multiplying a value that depended on the separation rate to an exponential-type TSL. The second one followed the framework of viscoelastic methods, claiming that rate-dependency was originated from phenomenon, including creep, relaxation and hysteresis characteristics, for periodically changing loads. More specifically, it was basically a generalized Maxwell model, including a Hooke element and a number of N Maxwell elements in parallel, as shown in Figure 25. The numerical investigation of uniaxial tension of a prismatic specimen (with a weak interface in the middle) showed that under stress relaxation, creep and cyclic loading conditions, the proposed viscoelastic model gave more realistic results.

Xu et al. [102,103] proposed a viscoelasticity cohesive zone model, which consisted of a rate-independent exponential CZM and a Maxwell model in parallel. Discussions of the proposed model showed that there existed a low limit and high limit for the discussed separation rate, meaning that at considerable low and high separation rates, they did not influence the mechanical behavior of the model much, while at the middle range of rates, the influence was pronounced. The model was then validated by comparing the experimental and numerical results of load-crack opening displacement of DCB under four constant loading speeds and a variable loading speed. The numerical results also provided insights into the correlation between the crack speed and the energy release rate of the thermoplastic adhesive.

### 3.2. Modeling of Rate-Dependent Bulk

**Figure 26:**
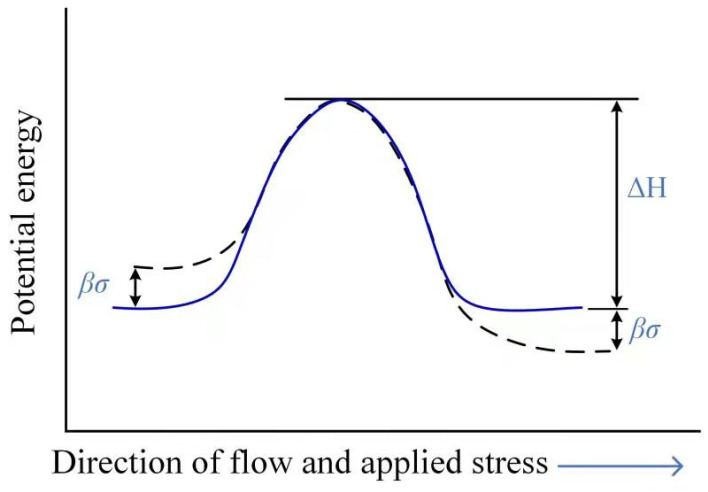
A schematic representation of the Eyring model.

The rate-dependency of bulk, which consisted of polymer, fiber and the interface in between, are mostly dominated by the time-dependent behavior of the polymer. Most works published in the literature focus on the rate-dependency of the amorphous polymer (e.g., polycarbonate and polymathylmethacrylate). The time-dependency of the polymer is also known as viscosity, which is normally dealt with in the viscoelastic model, the viscoplasticity model or a combination of both. These models could be either molecular-mechanism-based or phenomenological. At the molecular scale, the seminar work on the rate-dependent plastic flow in glassy, amorphous polymers was conducted by Eyring [104]. Eyring assumed that the deformation of a polymer was a thermally activated rate process involving the motion of segments of chain molecules over potential barriers [64], which could be described by non-linear dash-pot phenomenonically. The model proposed by Eyring includes parameters, such as activation energy and activation volume, which may uncover the underlying molecular mechanisms. Eyring assumed that macroscopic deformation resulted from basic processes that are either intermolecular (e.g., chain-sliding) or intramolecular (e.g., a change in the conformation of the chain), whose frequency depends on the ease with which a chain segment can surmount a potential energy barrier of height ΔH [64]. The schematic representation of the Eyring model is shown in Figure 26. The applied stress could disturb the dynamical equilibrium by causing linear shifts βσ of the energy barriers in a symmetrical way and changing the net flow frequency ν in the direction of the applied stress. Therefore, the rate of change of strain reads,

(7) $$\frac{de}{dt}=\dot{e}={\dot{e}}_{0}\exp\left(-\frac{\Delta H}{RT}\right)\sinh\left(\frac{\nu \sigma }{RT}\right)$$

where ${\dot{e}}_{0}$ is a constant pre-exponential factor; $\dot{e}$ is the strain rate; ΔH is the activation energy; σ is the applied stress; *R* is the universal gas constant; *T* is the absolute temperature and is the activation volume for the molecular event. Later developments of Eyring’s model by Robertson [105], Argon [106], Mulliken and Boyce [80] try to capture all observed phenomenon of the rate-dependent plastic flow in amorphous glassy polymers. Mulliken and Boyce [80] explored the connections between transitions in yield behavior and transitions in viscoelastic behavior, the mechanisms controlling these transitions and how the location of these transitions shift with both temperature and strain rate. Based on the Ree–Eyring yield theory for rate-activated processes, a kind of rate-, temperature-, and pressure-dependent finite-strain deformation constitutive model was proposed. The assumption made in the model was that the resistance to deformation could be decomposed into two parts: intermolecular resistance to chain-segment rotation (elastic spring and viscoplastic dash-pot) and entropic resistance to chain alignment (Langevin spring). A numerical simulation with FEM showed that the proposed constitutive model correctly predicted yield stress values, as well as the strain rate regime of the transition in the yield behavior for PC and PMMA.

**Figure 27:**
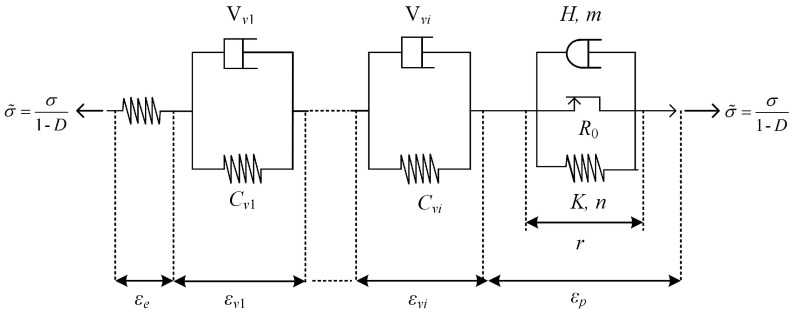
A schematic representation of the rheological scheme.

Macroscopic phenomenological models used to capture the viscosity of a polymer are usually linear viscoelastic models (Maxwell, Kelvin, standard linear solid model by Zener, Multiple-elements models), non-linear viscoelasticity models and viscoplastic models. Goldberg et al. [107] developed a viscoplastic state variable model based on the Bodner–Partom model, which was used for the analysis of metals above one-half of the melting temperature to describe the strain-rate-dependent deformation of the polymer. Praud et al. [108] proposed a phenomenological model for semicrystalline thermoplastic polymers, which accounts for viscoelasticty, viscoplasticity and ductile damage. As shown in Figure 27, this model consists one single linear spring, *N* viscoelastic Kelvin–Voigt branches (i.e., a linear spring and a linear dash-pot) assembled in parallel and a viscoplastic branch (i.e., a frictional element, a non-linear spring and a non-linear dash-pot both assembled in parallel) assembled in series. The damage is introduced based on the well-known principle of effective stress. A number of experiment tests have validated that the formulated model allows for characterization of viscoelasticity, viscoplasticity and damage of the semicrystalline thermoplastic polymers under complex and non-proportional loading paths.

**Figure 28:**
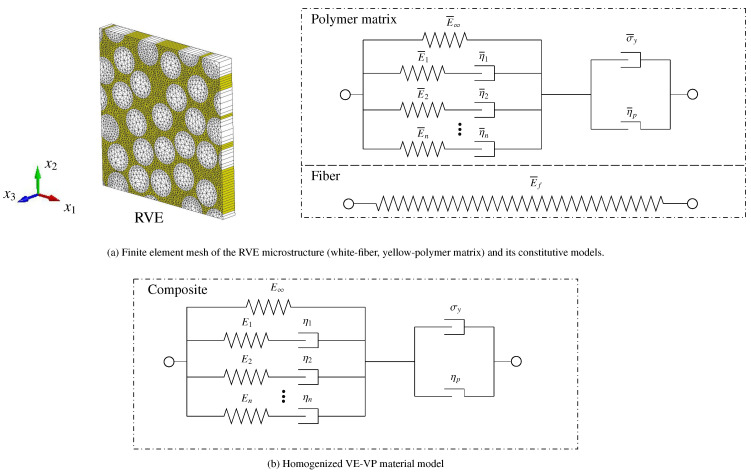
A comparison of the stress–strain relation of monotonic loading for six different strain rates between RVE simulation and the homogenized numerical model [116].

Fiber, as another phase of the composites, is usually considered to be a rate-independent and linear elastic constitutive model is usually adopted to describe its mechanical behavior. The debonding of fiber and the matrix, which might also have a remarkable influence on the overall response, could be treated as damage to the fiber. The interface model proposed by Needleman [109] could be used as an example, as shown in Allen et al. [110]. The rate-dependent traction-separation law introduced in Section 3.1 can be incorporated into the interface model to account for interfacial rate-dependency. A homogenization process is needed for polymeric composites that might contain aligned or randomly oriented discontinuous fibers since the matrix and discontinuous fibers have different material characteristics. This issue have already been addressed by Matzenmiller and Gerlach [111], Lissenden and Herakovich [112], Miled et al. [113], He et al. [114] and Yang et al. [115] and Liu et al. [116]. Liu et al. [116] proposed a step-by-step numerical homogenization procedure to calibrate a homogenized viscoelastic-viscoplastic (VE-VP) model with the same formulation as the VE-VP model used for describing the polymer behavior with a representative volume element (RVE)-based homogenization method. The stress–strain curves for the homogenized numerical model using the calibrated values are plotted in Figure 28 for six different cases from 0.00035/s to 0.175/s. It is shown that the homogenized model solution matches very well with the RVE simulation results for the studied strain rate ranges. With this numerical framework, a wide range of polymer matrices, which show viscoelastic-viscoplastic behaviors, can be considered by adapting the viscosity parameters of the polymer model.

### 3.3. Mirco-Scale and Meso-Scale Inertia Effects

Micro-scale and meso-scale inertia effects are both recognized to have contributions to the rate effects, so they should be considered in the numerical model. The heterogeneity of the microstructure of composite materials causes dispersion in wave propagation associated with dynamic loading. This dispersion phenomenon is a result of the local motion of the microstructure-driven bymultiple wave reflections occurring at the interfaces between the fiber and polymer matrix. The dispersion and attenuation observed in composites subjected to wave propagation should be reflected in the constitutive model to account for the effect of the microstructure. Achenbach et al. [117] proposed an “effective stiffness” theory by which the actual composite was transformed into a homogeneous higher-order continuum with a microstructure. Hegemier et al. [118] proposed mixture theories as models of the elastodynamics of composites, and the mixture interaction and constitutive relations for all constituents were properly accounted. Other higher-order homogenization theories, which normally introduce multiple spatial scales and higher order terms in the asymptotic expansion, could refer to Fish and Chen [119]. Besides, Fish et al. [77] developed a mathematical homogenization scheme in which micro-scale inertia effects are taken into account as an inertia-induced eigenstrain. The micro-scale equation of motion was solved by the perturbation expansion of displacement, inertia and weight functions. This model is adopted in Liu et al. [78], and the application of this method for fiber-reinforced composites is discussed. Karamnejad and Sluys [120] developed a continuous-discontinuous computational homogenization scheme to capture the micro-scale inertia effects based on the dispersive homogenization model proposed by Fish et al. [77]. At the meso-scale, the inertia effects could be captured by solving the equation of motion by the finite element method.

### 3.4. Thermo-Mechanical Model

**Figure 29:**
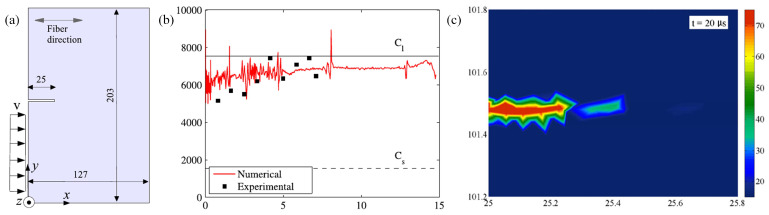
(**a**) Geometry and boundary conditions of a single-edge notch composite plate; (**b**) the crack speed of an intersonically propagating shear crack; (**c**) temperature field ahead of a notch due to fracture at a typical time instant [123].

Temperature effects could be properly considered following the coupled thermo-mechanical framework. Heat generation in bulk material and the fracture process zone should be considered properly with a constitutive model reflecting the influence of temperature. Bjerke and Lambros [82] proposed a thermomechanical dissipative cohesive zone model to predict the dynamic crack tip heating increase in brittle amorphous polymers. Ozdemir et al. [121] developed a model suitable for the analysis of material interfaces at different scales in which the concept of thermal damage was used to quantify the reduction in the heat flux across the interface. Zreid et al. [122] developed a thermomechanically coupled viscoelastic cohesive zone that used temperature relations in both traction laws and the viscosity of the dash-pot and a heat conduction law across the interface. The scheme is able to resolve the fully coupled simultaneous solution of the thermal field and the deformation field. Ahmed and Sluys [123] simulated the impaction test carried out by Coker and Rosakis [124] and validated that the localized increase in temperature in the crack-tip region can affect the crack propagation speed (see Figure 29). Heat generation during fracture and transfer through an interface is achieved by solving the equation of motion and the energy conservation equation simultaneously, accounting for the effects of cohesive interfaces, friction, inertia and heat conduction.

### 3.5. Characteristic Fracture Time

When damage was used for the interface model, it is important to evaluate the magnitude of characteristic fracture time to ensure correct temporal discretization within the numerical simulation. Camacho and Ortiz [48] calculated the characteristic fracture time by considering a spall plane with a co-linear array of flaws at x = 0, which is reached at t = 0 by an incident square pulse of magnitude σ and duration τ. An explicit expression for the pulse duration needed for the spall to occur is derived using the wave equation and assumed cohesive law. Guimard et al. [43] studied the dynamic propagation of mode-II delamination in composite laminates, and the characteristic fracture time was estimated using a steady-state 1D approach. Indeed, it corresponds to the ratio of the process zone length to the current crack velocity, leading to the equation:

(8) $$t_{c}^{cdm}=\frac{1}{n}\sqrt{\frac{3EhG_{IIc}\left(1-{\left(\frac{\dot{a}}{c_{L}}\right)}^{2}\right)}{8\dot{a}\tau_{0}^{2}}}$$

in which $\tau_{0}$ is the initiation stress threshold; $\dot{a}$ is the crack speed; *E* is the Young’s modulus; ρ is the mass density; *n* is a constant between 0.4 and 2; *h* is the height of the tested composite beam; $c_{L}=\sqrt{E/\rho }$ is the wave speed; $G_{IIc}$ is the mode-II fracture toughness for the tested CFRP material. In FE calculations with an explicit time scheme, the time step was defined to be lower than the calculated characteristic fracture time to ensure a predictive simulation of the dynamic process.

## 4. Conclusions

Strain rate effects in fiber-reinforced polymer composites can be divided into two categories: one is the rate-dependent deformation of composite before strain softening and the strain rate-dependent failure process inside the failure zone.

There exists inconsistent experimental results about the influence of the strain rate on the dynamic deformation properties (e.g., stiffness modulus, strength, ultimate strain) and the fracture energy of composites. Positive rate-dependency, rate-insensitive behavior or negative rate-dependency can be found depending on the composite system (the type of fiber and matrix), the test rate range, loading type (tensile, compressive, shear, flexura etc.) and load direction (longitudinal, transverse or off axis).

The underlying mechanisms of the observed rate-dependent deformation and the failure of composites are most likely to involve multiple length scales and time scales. They can be classified as: the viscosity of composite constituents, rate-dependency of the fracture mechanism, inertia effects at both mesoscale and microscale, characteristic fracture time and thermomechanical dissipation.

For numerical investigation of these mechanisms, the following numerical models or techniques are proposed: (1) classical time-independent material model, such as elasticity, plasticity and damage models, which are enriched with a viscosity formulation to describe the viscous composites; (2) rate-dependent cohesive zone models are developed such that the cohesive strength and fracture energy of the composites becomes strain-rate-dependent; (3) a micro-inertia-related formulation is incorporated into the equation of motion such that multiscale inertia is incorporated into the same numerical solution framework; (4) the time stepping of a numerical model is defined to be lower than the calculated characteristic fracture time to ensure a predictive simulation of the dynamic process; (5) thermomechanical numerical models are developed such that the heat generation, conduction and its interaction with the polymer composites are simulated. It should be noted that a comprehensive multiscale framework that incorporates all the mechanisms is still lacking.

## Data Availability

Not applicable.
